# A Comprehensive Survey of Privacy-Enhancing and Trust-Centric Cloud-Native Security Techniques Against Cyber Threats

**DOI:** 10.3390/s25082350

**Published:** 2025-04-08

**Authors:** Tuba Arif, Byunghyun Jo, Jong Hyuk Park

**Affiliations:** Department of Computer Science and Engineering, Seoul National University of Science and Technology (SeoulTech), Seoul 01811, Republic of Korea; tuba@seoultech.ac.kr (T.A.); jbh1020@seoultech.ac.kr (B.J.)

**Keywords:** cloud-native security, cyber security, privacy-enhancing security, trust management, zero-trust security, AI-driven threat detection, DevSecOps

## Abstract

Cloud-native architecture is becoming increasingly popular in today’s digital environment, driving the demand for robust security precautions to protect infrastructure and applications. This paper examines a variety of privacy-enhancing and trust-centric tools and techniques intended to meet the unique security requirements within cloud-native environments. Specifically, a variety of solutions are covered, such as runtime protection platforms for real-time threat detection and responses, cloud-native endpoint security solutions for ensuring trust and resilience in dynamic contexts, and service mesh technologies for secure service-to-service communication. Furthermore, we examine the roles of cloud-native encryption, cloud-native identity and access management, and container image scanning technologies in protecting containerized applications and preserving data privacy in transit and at rest. The importance of threat detection and response systems, cloud-native security information and event management (SIEM) solutions, and network security are also covered to strengthen trust and transparency in cloud-native security. We also present a thorough case study that demonstrates how security measures are applied across multiple layers, including application, network, infrastructure, and security, and compliance, to ensure holistic security in a cloud-native architecture. By investigating these privacy-enhancing methods and technologies, organizations may improve the security posture of their cloud-native implementations, reducing risks and ensuring the trustworthiness of their information and applications in the ever-changing ecosystem of today’s digital landscape.

## 1. Introduction

The concept of cloud-native architecture has become essential for efficiency, scalability, and resilience in today’s quickly changing digital environment, which is marked by fast technological improvement and unrelenting innovation. The use of cloud-native techniques has moved from being a choice to an imperative as companies fight to remain agile and competitive. This study seeks to explain the fundamentals of cloud-native architecture, highlight the vital importance of cloud-native applications security, and highlight the crucial role that cloud-native architecture plays in the fields of cybersecurity and artificial intelligence (AI) [1,2,3,4]. At its core, cloud-native design embodies an all-encompassing methodology for developing and implementing apps that is thoroughly developed to fully utilize cloud computing. Cloud-native applications are modular, scalable, and adaptive, in sharp contrast to traditional monolithic infrastructures, which are distinguished by their inflexible, tightly connected frameworks [5,6]. They are designed with a microservice architecture, which breaks down large programs into smaller, loosely linked parts that run in separate containers. Because each component can be updated, scaled, and deployed individually, this modular architecture encourages fast innovation and continuous delivery within the development community.

Cloud-native architecture’s emphasis on containers and orchestration tools like Kubernetes is fundamental to its conception. The fundamental units of cloud-native apps are containers, which contain the application logic together with its dependencies, guaranteeing consistency between development and production environments. Meanwhile, cloud-native solutions can run smoothly along a scale due to Kubernetes, a powerful orchestration platform that performs the deployment, scaling, and maintenance of containerized apps [7,8,9]. Kubernetes and containers work together to provide the foundation of contemporary cloud-native systems, enabling businesses to attain previously unheard-of levels of resilience, efficiency, and agility [10,11,12,13,14]. The increasing integration of cloud-native frameworks with advanced security practices, such as automated compliance checks, real-time threat intelligence, and proactive risk management systems, represents a significant evolution in the landscape of cloud security [15,16]. These emerging trends not only enhance the security of cloud-native environments but also address the increasing sophistication and frequency of cyber threats. Still, there are difficulties associated with the shift to cloud-native technology, especially concerning security [17]. The goal of cloud-native security is to strengthen cloud-native infrastructure and applications against a constantly changing set of threats and vulnerabilities using a broad range of techniques, methods, and technologies [18]. Conventional security paradigms are inadequate in light of the dispersed nature of cloud-native settings and the dynamic nature of containers and microservices. As a result, companies need to adopt a comprehensive and flexible security posture that covers the whole software development lifecycle, from conception to execution [19]. This includes the adoption of emerging technologies such as AI-driven security enhancements and blockchain-based transaction systems, which provide robust defenses against the dynamic threats inherent in cloud-native architectures.

The defense-in-depth concept, which promotes the deployment of several levels of security measures to successfully reduce threats, is at the core of cloud-native security. This includes encrypting data in transit and at rest to maintain confidentiality, putting strong identity and access management (IAM) mechanisms in place to control resource access, protecting the developing process to prevent the deployment of vulnerable code, and closely monitoring and logging activities to quickly identify and address security incidents in real time [20,21]. Furthermore, specialized container security solutions are essential for recognizing and mitigating security risks specific to containerized settings. Examples of these include image scanning and runtime protection. When it comes to artificial intelligence, cloud-native architecture is a driving force behind the creation and implementation of AI-driven services and applications [22,23]. Because cloud-native platforms are naturally scalable and flexible, they are well suited to handle the computational needs of AI workloads, which frequently require large amounts of computing and storage [24,25]. Organizations may accelerate the creation and implementation of AI models by utilizing cloud-native platforms. This allows them to leverage machine learning and data analytics to stimulate creativity and extract valuable insights.

Furthermore, given the growing sophistication of cyber threats, cloud-native architecture plays a critical role in enhancing cybersecurity capabilities. Organizations face difficult task in protecting their data and systems from a variety of threats, including supply chain vulnerabilities, ransomware, and malware, because of the increasing number of digital assets and the growing complexity of IT infrastructure [26,27,28]. The incorporation of predictive security measures, leveraging real-time data and adaptive response strategies, represents a proactive approach to cloud-native security, which is critical to addressing these challenges effectively. Given this, cloud-native security stands out as the cornerstone of contemporary cybersecurity tactics, providing a proactive and flexible method of threat identification and elimination. It is impossible to overestimate the importance of cloud-native security in the current environment. It is more important than ever to protect cloud-native systems from emerging threats and vulnerabilities as organizations are depending more and more on these technologies to foster creativity and agility. The wide-ranging effects of a data leak or security event might include monetary losses, harm to one’s reputation, and regulatory attention [29]. As a result, making significant investments in strong cloud-native security protocols is not only a wise commercial move, but also a crucial necessity for protecting the confidentiality, availability, and integrity of data and applications.

Hence, cloud-native architecture represents a fundamental change in the way that applications are developed, deployed, and managed in the cloud environment. Cloud-native architecture, which prioritizes modularity, scalability, and resilience, enables enterprises to innovate quickly, grow in real time, and adapt quickly to changing market conditions. As cloud-native security technologies continue to evolve, driven by advances in AI and machine learning, blockchain, and integrated security operations centers, they pave the way for even more resilient and secure digital infrastructures. To fully utilize cloud-native systems, however, security must be given careful consideration and supported by proactive steps to identify, reduce, and address security threats throughout the software development lifecycle. Organizations may access the full range of advantages offered by cloud-natives while reducing the inherent security problems by adopting cloud-native security principles and best practices. This will pave the way for a more resilient and secure digital future.

### 1.1. Motivation

Research on securing cloud-native apps in real-world environments must continue as both technology and cyber threats are always evolving. Cloud-native apps, which are based on microservices, containers, and orchestration platforms, bring new challenges that need further cybersecurity research and development. Because technology is advancing so quickly, one needs to be continually researching to stay ahead of emerging threats. Cloud-native systems leverage contemporary technology like orchestration tools such as Kubernetes and containerization. As these technologies advance, new attack vectors can surface, requiring researchers to conduct analysis, comprehend issues, and employ alternative creation to mitigate any risks. Furthermore, the distributed and resilient architecture of cloud-native systems necessitates unique security solutions. The flexibility of applications to scale dynamically across many cloud providers and geographical regions sometimes complicates the administration of identity, access control, and inter-service communication security. Researchers need to go deep into these difficulties to propose practical security solutions that take into consideration the special qualities of cloud-native applications. Furthermore, the incorporation of third-party services and dependence on open-source components in cloud-native ecosystems introduce additional levels of complexity and potential security vulnerabilities. Further research is required to evaluate these external services’ security postures, understand their potential impact on the overall security of the application, and implement robust security controls in a real-world environment.

To fend off attackers in the face of sophisticated cyber threats, one must adopt a proactive and research-based approach. Since hackers are always changing and coming up with new tactics, security specialists need to look at cutting-edge methods for threat detection, incident response, and legal evaluation that are specific to cloud-native environments. Businesses may establish strong security postures that can effectively manage crises and withstand evolving threats with the help of this study. Furthermore, ongoing research is necessary to ensure that cloud-native applications comply with the most recent rules, as legal and compliance standards are subject to change over time. Researchers play a critical role in supporting businesses in evaluating and understanding legislative changes as well as designing controls that enhance the overall security posture of cloud-native deployments and meet compliance requirements. Therefore, further study is required to protect cloud-native applications in real-world scenarios because of the intricate relationships among emerging technologies, dynamic cloud architectures, and shifting threat environments. Continuous research ensures that security policies are effective, adaptable, and capable of managing the ever-changing challenges that occur during the creation and deployment of cloud-native apps. Through persistent exploration and ingenuity, organizations may establish and maintain robust security measures to protect their assets, data, and operations on the cloud.

### 1.2. Contribution

This study offers several key contributions to the field of cloud-native security:

- We provide a detailed survey of unique security challenges, including the dynamic nature of microservices, container vulnerabilities, and complexities in decentralized access control in cloud-native environments.
- We offer a comprehensive analysis of current security tools and techniques, including runtime protection platforms, DevSecOps pipelines, cloud-native security information and event management (SIEM), and IAM systems.
- We examine the integration of advanced technologies such as AI-driven threat detection and blockchain-based access control as innovative solutions for cloud-native security.
- We present a case study illustrating security solutions applied across multiple layers, including application, network, infrastructure, and security and compliance, highlighting how these measures ensure a consistent security posture throughout cloud-native solutions.
- Lastly, we summarize the future research directions by proposing a framework for developing adaptive security measures, advanced threat detection techniques, and robust access control mechanisms tailored to cloud-native environments.

Thus, this paper serves as a thorough resource for developers and researchers looking to understand the evolving security landscape of cloud-native applications and suggests a path for future research.

### 1.3. Organization

This study unfolds various cloud-native amenities, security aspects, applications, as well as securing techniques in the cloud environment. The rest of the paper is structured as follows. We examine the relevant literature in Section 2. Section 3 introduces cloud-native applications along with an explanation of their strategies. A thorough approach for securing cloud-native applications is provided in Section 4. Section 5 presents a detailed case scenario that demonstrates the application of security measures in a real-world scenario. Ultimately, Section 6 addresses the obstacles and solutions for the future path, while Section 7 concludes the paper.

## 2. Related Works

**Figure 1:**
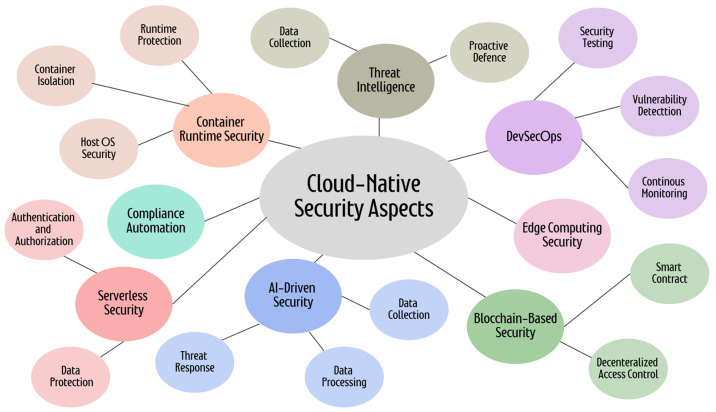
A structured overview of key cloud-native security aspects, illustrating focus areas such as container runtime security, DevSecOps, blockchain-based mechanisms, AI-driven threat detection, and compliance automation.

A current paradigm change in the conception, creation, implementation, and administration of software applications inside the cloud computing framework is being referred to as “cloud-native amenities”. This method maximizes the efficiency, scalability, performance, and availability of applications by utilizing the built-in features of cloud infrastructure. Fundamentally, as illustrated in Figure 1, cloud-native services and security aspects comprise a collection of ideas and procedures that facilitate the development of extremely scalable, portable, and resilient applications. The core of these amenities is containerization, which isolates and lightweights’ individual application components along with their dependencies inside separate containers. Containerization assures consistent behavior across many settings, expedites deployment, and makes efficient use of the resources possible [30].

Several research works have tackled the difficulties and developments in cloud-native application security, offering significant perspectives in the domain. In [31,32], the author examined the security implications of cloud-native environments’ dynamic nature and the necessity for adaptable security solutions that can keep up with containerized architectures’ quick changes. Similarly, in their investigation of the security consequences of microservice architectures in cloud-native environments, researchers in [33,34] emphasized the need for strong authentication techniques and decentralized access control systems to reduce the danger of unwanted access. DevSecOps pipelines have received an enormous amount of attention in recent studies when it comes to security solutions. To support a security-first approach to cloud-native application development, the author [35,36,37] investigated the function of DevSecOps pipelines in automating security testing and detecting vulnerabilities across the software development lifecycle. Furthermore, researchers in [38,39] examined how cloud-native threat intelligence platforms may be integrated into DevSecOps pipelines to help enterprises proactively fight against cyber threats by gathering and evaluating threat data from various sources.

Moreover, research efforts have focused on improvements in container runtime security. In their investigation into the difficulties of protecting containerized apps from runtime flaws, researchers in [40,41] put forth cutting-edge methods for container isolation and host OS vulnerability mitigation. Similarly, to improve the security posture of cloud-native systems, researchers in [42,43,44,45] investigated the use of sandboxing approaches and sophisticated runtime protection procedures. Additionally, research has concentrated on privacy concerns and the automation of compliance in cloud-native contexts. The difficulties of maintaining compliance with security laws and regulations, such as the CCPA and GDPR, in cloud-native applications were investigated in studies by the author [46,47]. The study highlighted how crucial it is to put strong data encryption and access control systems in place to secure private user data and reduce legal and reputational liabilities.

Apart from this, the industry has furnished significant perspectives on realistic obstacles and optimal approaches for protecting cloud-native applications. Prominent cybersecurity companies, like Palo Alto Networks and FireEye, have released reports that highlight new risks and offer security recommendations for enterprises converting to cloud-native systems [48,49]. Examples of how businesses can use cloud-native security tools and practices to improve their security posture are provided by the case studies on effective cloud-native security implementations, including those included in the documentation for AWS and Microsoft Azure. Moreover, cloud-native security research and development have advanced greatly as a consequence of cooperative efforts and open-source projects. Security researchers and business experts have collaborated on projects like Cloud-Native Computing Foundation (CNCF) Security [50] and the Kubernetes Security Audit to find and fix security flaws in cloud-native technologies [51]. Furthermore, the capacity to offer runtime security tracking and container image scanning in cloud-native systems has made open-source security solutions like Falco and Clair increasingly popular [52,53].

Furthermore, recent studies have looked at how cutting-edge technologies like blockchain and artificial intelligence interact with cloud-native security. Investigations into the potential of AI-driven security solutions for threat detection and mitigation in cloud-native systems were conducted by the authors in [54,55]. This research addressed the analysis of security telemetry data and the identification of unusual behaviors indicative of cyber risks through the application of machine learning techniques. Furthermore, in recent years, blockchain-based methods of cloud-native security have become more popular. A decentralized access control system utilizing blockchain technology was presented by researchers [56,57,58,59,60] in their research to improve security and transparency in microservice-based architectures. The system attempts to mitigate the risks of unauthorized access and single points of failure by scattering access control policies throughout a blockchain network, tackling important issues in cloud-native application security.

Additionally, research has looked at how business standards and legal frameworks affect cloud-native security procedures. An author investigated how cloud-native security practices fit within well-known frameworks like ISO/IEC 27001 [61,62] and the NIST Cybersecurity Framework. To guarantee compliance with legal requirements and industry’s best practices, the study emphasized how crucial it is to incorporate security controls and legal requirements into cloud-native development procedures. Furthermore, the importance of a DevSecOps culture and practices in boosting cloud-native security has also been the subject of recent studies. The integration of security practices into DevOps workflows was the subject of studies by authors [63,64]. These studies emphasized the significance of collaboration between security, operations, and development teams to maintain continuous security throughout the software development lifecycle. The advantages of implementing a DevSecOps methodology, including increased agility, a quicker time-to-market, and a stronger security posture in cloud-native environments, were emphasized in this research.

Additionally, research has investigated the benefits and difficulties associated with protecting particular cloud-native technologies, like edge computing and serverless computing. Strong authentication, authorization, and data protection procedures are essential for serverless applications, according to research by an author in [65], which examined security implications for serverless architectures. Comparably, research by authors in [66,67] examined edge computing environments’ security issues and best practices, highlighting how crucial it is to secure edge devices, data transfer, and app deployment in decentralized edge environments. Additionally, the analysis of vendor landscapes, market trends, and adoption trends for security systems in cloud-native environments has offered insightful information on the current level of cloud-native security procedures and trends. These studies provide enterprises looking to improve their cloud-native security posture and successfully manage the constantly changing threat landscape with useful guidance as demonstrated in Table 1.

Thus, this study attempts to provide a thorough overview of the state of the art of cloud-native security research and suggests opportunities for further research by integrating insights from studies, industry publications, case studies, and collaborations. This paper aims to add to the wealth of information on securing cloud-native apps and to clarify the most recent developments and industry best practices for practitioners and researchers through an examination of the existing collection of the current literature and real-world examples.

## 3. Cloud-Native Applications

**Figure 2:**
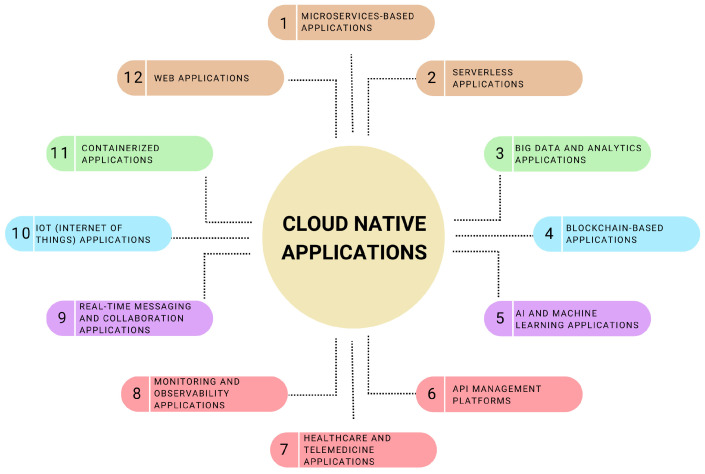
The categorization of prominent cloud-native application types across diverse domains, such as AI, big data, IoT, blockchain, and healthcare systems.

Utilizing every aspect of cloud computing to achieve unmatched efficiency, scalability, and flexibility, the concept of cloud-native apps represents a paradigm shift in the development, deployment, and operation of software. Now, let us explore the various kinds of cloud-native applications in more detail, as shown in Figure 2, to take a closer look at their practical uses.

- *Microservice-based applications:* Microservice architecture is a methodological advancement in software design that consists of distinct, self-contained services that communicate over well-defined Application Programming Interfaces (APIs) and carry out specialized tasks within systems. Teams may independently create, implement, and scale portions of the application with this architecture, which improves agility and shortens time-to-market. As exemplary cases, consider Netflix, Spotify, and Airbnb, where every microservice handles a different function or service, such as listing administration, user authentication, or video streaming, enabling these platforms to quickly develop and adjust to shifting consumer demands [68,69].
- *Web applications:* Among the most well-known and often utilized categories of cloud-native applications are web applications. These cloud-native applications provide high availability and scalability and may be accessed from any location using a web browser. Web applications developed with a cloud-native strategy can scale dynamically in response to user demand, ensuring performance and cost-effectiveness. Cloud-native web applications are best demonstrated by e-commerce sites like Amazon and eBay, which manage millions of transactions and customer interactions with ease [70]. Similar to this, cloud-native architectures are used by social media global giants like Facebook and Twitter to dynamically distribute content to billions of users globally, demonstrating the capacity to manage enormous volumes of data and user connections in real time.
- *Serverless applications:* With serverless computing, the cloud provider manages the execution environment, scales, and bills based on actual resource consumption, freeing developers to concentrate on building code that supports business logic. This technique works especially well with event-driven systems, in which programs react to certain occurrences, including file uploads, hypertext transfer protocol (HTTP) requests, or modifications to databases. An example of how serverless computing facilitates quick development and deployment cycles is the ability to create apps that scale automatically and economically with AWS Lambda, Azure Functions, and Google Cloud Functions [71,72].
- *Containerized applications:* Docker is a prime example of containerization technology that has transformed application deployment by encapsulating programs within lightweight containers. The application code, libraries, and dependencies are all included in this encapsulation, which assures consistency between various computer systems. Microservice architectures depend on containerized applications because they are cloud-native by nature and provide scalability, isolation, and simplicity of deployment. The orchestration solution for containers, Kubernetes, significantly improves containerized application administration by facilitating smooth rollouts of updates or new features, auto-scaling, and self-healing [73].
- *Big data and analytics applications:* Modern digital technologies need to handle and analyze massive datasets quickly and efficiently due to the proliferation of data. Cloud-native applications use cloud-based big data systems to process large amounts of data, such as Google BigQuery, Apache Spark, and Hadoop [74]. These platforms provide the scalability and flexibility needed for data ingestion, storage, processing, and visualization. By offering insights that drive innovation and decision-making, they support a broad range of applications, from genomic sequencing and scientific research to business intelligence and customer analytics.
- *IoT (Internet of Things) applications:* IoT applications use cloud computing to gather, handle, and evaluate data from networked sensors and devices in a variety of settings, including cities and factories as well as households. Because of the cloud’s enormous processing power and scalability, these applications frequently need real-time processing and analytics to produce actionable insights. To process and manage the massive influx of data from IoT devices and enable advanced features like maintenance forecasting and personalized user experiences, cloud-native technologies are essential to smart home systems, which include security cameras and thermostats, as well as industrial monitoring systems and connected vehicle solutions [75,76,77].
- *Blockchain-based applications:* Blockchain technology leverages cloud infrastructure’s scalability, security, and dependability to host decentralized apps (DApps). Utilizing the immutable and transparent properties of blockchain technology within a cloud-native framework, these applications, which range from cryptocurrency exchanges to supply chain tracking systems and decentralized finance (DeFi) platforms, offer new methods for managing data, decentralized from centralized control, and conducting transactions [78].
- *Real-time messaging and collaboration applications:* Real-time communication and collaboration tools, which need to be highly available, scalable, and latency-free to offer flawless user experiences, are best supported by cloud-native architecture. Cloud-native applications can facilitate immediate global communication and collaboration, revolutionizing the way people work and interact [79,80]. Examples of such applications include messaging apps such as WhatsApp, videoconferencing applications like Zoom, and collaborative document editing tools like Google Docs.
- *AI and machine learning applications:* Cloud-native technologies are being used in a novel way by AI and machine learning applications, which make it possible to handle large datasets at scale and perform intricate computations. Large datasets require a lot of resources and time in order to train machine learning models. Cloud platforms provide specialized equipment, like GPUs and TPUs, for this purpose [81]. Cloud scalability is used by cloud-native AI applications, like Netflix and Amazon’s personalized recommendation engines, image and speech recognition services, and predictive analytics for business intelligence, to adjust to changing workloads and data volumes. This allows for the quick development and implementation of AI models.
- *Monitoring and observability applications:* Monitoring and observability are essential for assuring application performance and reliability in the complex environment of cloud-native apps. Tools that offer the insights required to identify and fix problems in distributed systems include Grafana for visualization, Prometheus for monitoring, and Elasticsearch for logging and tracing. By gathering and examining metrics, logs, and traces from different areas of a cloud-native application, these programs help developers and operators better comprehend system behavior, maximize performance, and preserve system integrity [82,83,84].
- *API management platforms:* The fundamental components of digital transformation are APIs, which allow software programs to exchange information and interact. Apigee, API Gateway, and Kong are examples of cloud-native API management programs that offer a scalable environment for API creation, management, and security [85]. These platforms provide developer interfaces for API discovery and collaboration along with the ability to monitor API usage, enforce access controls, and manage API traffic. These platforms facilitate innovation and integration across digital ecosystems by enabling enterprises to speed the creation of applications and services by exploiting cloud-native capabilities.
- *Healthcare and telemedicine applications:* Cloud-native apps, such as electronic health records (EHR), telemedicine, and remote patient monitoring, are revolutionizing patient care in the healthcare industry [86]. Strong compliance with health data laws, such as HIPAA, is necessary for these applications, which make use of the cloud’s capacity to handle and securely store sensitive data, enable real-time patient-provider communication, and support advanced analytics for diagnostic imaging and personalized medicine. Concisely, Table 2 provides a comprehensive analysis of these applications, techniques of implementation, and their effects on cloud-native amenities in the various sectors discussed in this section.

Hence, the impact of cloud services on many businesses and sectors is exemplified by the variety of cloud-native application types available. By utilizing the power of the cloud, companies and organizations can innovate, grow, and adapt in ways that were not possible before. This promotes digital transformation and allows them to provide improved services and solutions to satisfy the changing demands of their clients and other stakeholders.

## 4. Techniques for Securing Cloud-Native Applications

**Figure 3:**
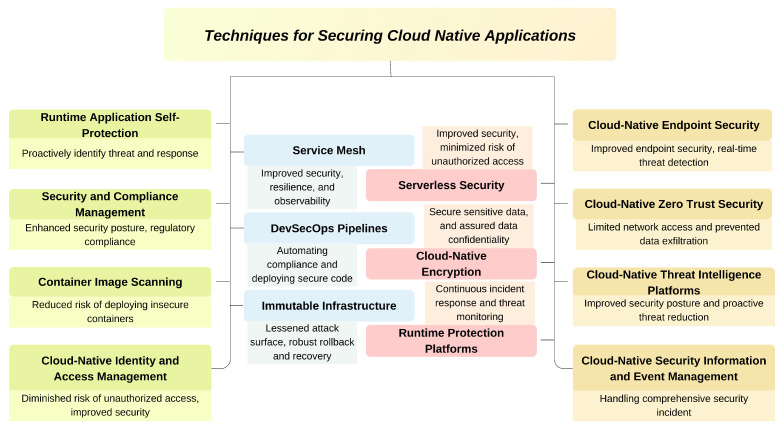
Overview of comprehensive security techniques for cloud-native applications, covering infrastructure hardening, identity and access control, data encryption, and advanced threat detection.

Strong security measures are more important than ever in the quickly changing world of cloud-native applications. Organizations are now able to strengthen their defenses and prevent a wide range of cyber-attacks due to recent developments in tools and strategies that handle certain challenges presented by cloud-native environments. These tools and methods, encompassing everything from innovative container security solutions to state-of-the-art threat intelligence platforms, offer comprehensive security coverage, as illustrated by Figure 3, throughout the software development lifecycle.

### 4.1. Overview of Security Techniques for Cloud-Native Applications

#### 4.1.1. Service Mesh

In cloud-native architectures, service mesh technologies like Istio, Linkerd, and Consul [87] have become more popular for controlling the intricacy of service-to-service communication. They offer a specialized infrastructure layer that manages load balancing, traffic routing, and service discovery in the context of inter-service communication. Mutual Transport Layer Security (mTLS) encryption, which guarantees safe communication between services by encrypting traffic and enabling both client-side and server-side authentication, is one of the primary security advantages provided by service mesh. To increase application resilience and reliability, service mesh platforms provide sophisticated traffic management features like circuit breaking and retry in addition to encryption. Additionally, they offer strong observability features, such as distributed tracing and metrics gathering, which enable operators to better understand service behavior and identify problems.

Service mesh improves security by enforcing security policies uniformly across microservices and centralizing communication controls by abstracting away communication-related issues from application codes. Additionally, it permits fine-grained access control, which lessens the likelihood of unwanted access and lessens the effects of security breaches by enabling operators to specify access policies based on service identity and role. All things considered, service mesh technologies are essential for improving the security posture of cloud-native apps since they offer an extensive feature set for effectively and safely handling service-to-service communication.

#### 4.1.2. Runtime Application Self-Protection (RASP)

Cloud-native settings require runtime security monitoring and protection. Aqua Security’s Trivy and Sysdig Secure [29] are two examples of runtime application self-protection (RASP) methods that meet this demand. RASP functions within the application runtime, enabling it to observe application behavior in real time and react to security risks dynamically, in contrast to typical application security tools that concentrate on perimeter protection. Moreover, to find and reduce security risks during application execution, RASP solutions usually include several methodologies, such as behavioral analysis, anomaly detection, and vulnerability scanning [88]. To keep an eye on containerized workloads and enforce security standards at runtime, they interact with container orchestration technologies like Docker Swarm and Kubernetes.

The capacity of RASP to offer granular security controls, catered to the unique needs of cloud-native applications, is one of its main features. RASP, for instance, can identify and stop malicious activity in containerized systems, like code injection and command execution, which lowers the attack surface and stops vulnerabilities from being exploited. Moreover, RASP systems give operators real-time access to the application security posture, enabling them to keep an eye on security events, look into issues, and quickly address risks. RASP helps enterprises to efficiently manage security risks and defend cloud-native apps against evolving attacks by providing proactive threat detection and response.

#### 4.1.3. Serverless Security

Cloud-native application development and deployment can be made easier by using serverless computing platforms like AWS Lambda and Azure Functions [89], which do not require infrastructure management. However, certain security issues such as function isolation, runtime monitoring, and access control are brought about by serverless systems. These issues are addressed by serverless security solutions, such as AWS Lambda Layers and Azure Functions Proxies, which offer several capabilities for protecting serverless apps. For instance, companies are capable of packaging access control policies and encryption libraries with serverless functions using AWS Lambda Layers, guaranteeing uniform security enforcement across deployments.

Similarly, Azure Functions Proxies improve security posture and reduce risks related to unauthorized access and misuse by enabling enterprises to establish rate limitations, fine-grained access controls, and authentication procedures for serverless functions. Runtime monitoring, which tracks function execution, resource usage, and application behavior in real time to quickly identify and address security events, is another essential component of serverless security. Operators can learn more about application performance and security posture by utilizing the runtime telemetry data collection and analysis capabilities provided by serverless security solutions. All things considered, serverless security solutions are essential for protecting cloud-native apps since they offer an extensive feature set for handling security threats related to serverless computing platforms.

#### 4.1.4. Security and Compliance Management

Centralized security management for cloud-native infrastructure and applications is provided by cloud-native firewall solutions like Palo Alto Networks’ Prisma Cloud and Aqua’s Cloud-Native Security Posture Management (CSPM) [90]. These solutions offer an extensive feature set to defend cloud-native settings from malware, network attacks, and data breaches, among other threats. As a result, network segmentation, which enables enterprises to separate workloads, apps, and data to lessen the effect of security breaches, assists enterprises in enforcing security boundaries and preventing unwanted access to critical resources by putting network policies and access restrictions into place.

Advanced threat detection and prevention features, including malware scanning, behavioral analysis, and intrusion detection and prevention systems (IDPSs), are also provided by cloud-native solutions. Governance, Risk, and Compliance (GRC) can lower the risk of security incidents and data breaches by detecting and blocking suspicious activity in real time by monitoring network traffic and application behavior. Moreover, compliance monitoring and reporting tools offered by cloud environments let businesses evaluate their security posture in comparison to industry’s best practices and legal requirements. Cloud-native firewall environments generate audit logs, compliance reports, and security dashboards to assist enterprises to prove they follow laws like GDPR, HIPAA, and PCI DSS. All things considered, cloud-native firewall solutions are essential for protecting cloud-native environments since they offer an extensive feature set that shields infrastructure and applications from a variety of threats.

#### 4.1.5. Immutable Infrastructure

Cloud-native environment creation and deployment can be facilitated by using infrastructure-as-code (IaC) templates via immutable infrastructure techniques. Organizations can automate infrastructure provisioning, configuration, and deployment with tools like HashiCorp Terraform and AWS CloudFormation [91,92], which guarantee consistency and dependability across environments. Reducing the attack surface by avoiding configuration drift and enforcing consistent deployment methods is one of the main advantages of immutable infrastructure. Organizations can lower the risk of misconfigurations and vulnerabilities by describing infrastructure configurations as code, which guarantees that infrastructure components are deployed predictably and securely.

Furthermore, in the event of a security issue or failure, immutable infrastructure enables quick rollback and recovery. Organizations may simply replace compromised components with known-good configurations by considering infrastructure as disposable, which minimizes downtime and lessens the impact of security breaches. Organizations can also optimize resource management and assure service sustainability by implementing predictive autoscaling, such as the PreVA framework, which lowers service disruptions while maintaining a high resource usage [93]. Infrastructure updating and auditing are supported by immutable infrastructure, which is an additional benefit. Organizations can ensure accountability and compliance with industry best practices and regulatory standards by tracking and auditing changes to their infrastructure by keeping a history of such modifications and configurations. All things considered, immutable infrastructure principles are essential to improving the security posture of cloud-native environments because they offer a dependable and uniform basis for managing and deploying infrastructure and applications.

#### 4.1.6. DevSecOps Pipelines

DevSecOps pipelines enable automated security testing, vulnerability scanning, and compliance checks by seamlessly integrating security practices into the software development lifecycle. Snyk, Fortify, and SonarQube are a few examples of tools that make it easier to integrate security checks at every step of the continuous integration and delivery (CI/CD) pipeline [94]. This lowers the risk of releasing unsafe code into production and encourages security-first development in cloud-native environments. Static code analysis, container image scanning, penetration testing, and other security checks and tests are frequently included in DevSecOps pipelines to find and fix security flaws early in the development process. Organizations may automate security testing and validation by integrating security into the CI/CD pipeline. This allows developers to find and address vulnerabilities before they are implemented into operational environments.

Support for compliance automation, which entails the application of security rules and legal requirements at every stage of the software development lifecycle, is another essential component of DevSecOps pipelines. Organizations may create and enforce compliance requirements as code with the help of tools like Terraform Compliance and Chef InSpec, which guarantee that infrastructure and applications comply with industry norms and security standards. IaC principles are also used by DevSecOps pipelines to deploy and configure cloud resources securely. Developers can now describe infrastructure configurations in code by using technologies like AWS CloudFormation and HashiCorp Terraform, which make it possible to install safe infrastructure consistently and repeatedly across cloud environments.

Furthermore, by including security controls in the deployment process, DevSecOps pipelines guarantee that applications are delivered safely and configured under industry standards. Organizations can identify and address security issues instantly with the help of tools like AWS Config and Azure Security Center, which offer continuous monitoring and remediation capabilities. All things considered, cloud-native environments greatly benefit from the culture of security and compliance that is generated by DevSecOps pipelines. DevSecOps pipelines enable enterprises to design and deploy protected applications with confidence by automating security testing, vulnerability scanning, and compliance checks. This lowers the risk of security breaches and ensures the integrity of cloud-native deployments.

#### 4.1.7. Cloud-Native Identity and Access Management (IAM)

In contemporary application designs, IAM solutions like AWS IAM, (role-based access control) RBAC IAM, and (attribute-based access control) ABAC IAM services are essential for protecting user access to cloud resources [95]. Organizations may reliably implement security rules by using these solutions, which offer centralized administration of user identities, access restrictions, and permissions across distributed environments. The ability to set restricted rights based on user classifications, groups, and asset properties is one of the main advantages of cloud-native IAM. This lessens the possibility of data exfiltration and security breaches by lowering the risk of illegal access and privilege escalation.

Multi-factor authentication (MFA) is another feature that cloud-native IAM systems provide [96]. This allows enterprises to improve security by requiring users to authenticate their identity with a biometric or one-time passcode before accessing sensitive resources. Even if credentials are compromised, MFA helps to prevent unwanted access by introducing an additional degree of authentication. Moreover, identity federation is made easier by cloud-native IAM solutions, which enable enterprises to link with external identity providers (IdPs) and build trust connections for user authentication across domain boundaries. This ensures compliance with security rules and regulatory requirements while providing users with seamless access to cloud services, regardless of the source of their authentication.

Apart from controlling access and authentication, cloud-native IAM solutions provide functionalities for auditing, monitoring, and reporting cloud resource access. This entails creating access logs, keeping an eye on user behavior, enforcing security regulations, and facilitating the tracking and analysis of access patterns by companies for incident response, forensic investigation, and compliance. All things considered, cloud-native IAM solutions are essential for protecting cloud-native applications because they offer an extensive feature set for controlling user identities, implementing access restrictions, and keeping an eye on access to sensitive resources in scattered environments.

#### 4.1.8. Container Image Scanning

To secure containerized applications, vulnerability detection and remediation in container images before deployment are critical tasks for container image scanning technologies like Anchore Engine, Docker Security Scanning, and Clair [97]. These solutions assist enterprises in lowering the risk of deploying unsafe containers in production settings by automatically analyzing container images for existing security risks, software misconfigurations, and legal concerns. Vulnerability detection, which entails checking container images for known vulnerabilities in software applications and dependencies, is one of the main functions of container image scanning technologies. Prioritizing and fixing high-risk vulnerabilities before launching containerized apps allows enterprises. This includes flaws reported in common vulnerability databases (CVEs), security alerts, and vendor-supplied modifications.

Tools for scanning container images can also feature compliance scanning, which enables businesses to enforce security guidelines and legal requirements for container images in addition to detecting vulnerabilities. This entails checking sensitive data, verifying image configurations, and guaranteeing adherence to industry standards like Docker Security Best Practices and Center for Internet Security (CIS) benchmarks. Moreover, the software development lifecycle may be seamlessly integrated with container image scanning solutions because of their interface with container registries and CI/CD pipelines. As a result, businesses can guarantee that only safe and compliant images are sent to production environments by automating the security testing and validation of container images as part of the development and deployment process.

Moreover, visibility and reporting features like dashboards, reports, and notifications are offered by container image scanning systems, enabling companies to track and keep an eye on the security posture of container images over an extended period. For containerized applications, this makes proactive risk management and ongoing security practice improvement possible. All things considered, cloud-native application security is greatly enhanced by container image scanning tools, which help enterprises identify and address security flaws in container images before deployment. This lowers the possibility of security breaches and guarantees the dependability and integrity of workloads that are containerized.

#### 4.1.9. Cloud-Native Encryption

Centralized security management for cloud-native infrastructure and applications is provided by cloud-native environments may encrypt data, both in transit and at rest, with the help of cloud-native encryption technologies like HashiCorp Vault, IAM Key Management Service (KMS), and Google Cloud Key Management Service (KMS) [98]. These solutions enable businesses to protect confidential information across distributed systems by providing a variety of encryption algorithms, key control features, and integration choices. Encryption at rest, or protecting data from unwanted access by encrypting them in file systems, databases, and object storage, is a fundamental component of cloud-native encryption solutions. This entails managing encryption keys securely to prevent unwanted decryption and encrypting data through cryptographic algorithms like RSA (Rivest–Shamir–Adleman) and AES (Advanced Encryption Standard) [99]. Beyond traditional cryptographic algorithms, other domains such as multimedia privacy have adopted hybrid approaches that combine compression and encryption to enhance data protection, as demonstrated in image privacy frameworks using DCT and nonlinear dynamics [100].

Apart from encryption at rest, cloud-native encryption methods provide encryption in transit, which encrypts data while they are transferred between various cloud-native environment components and services. This involves encrypting data communications and guaranteeing confidentiality and integrity while they are in transit by employing protocols like TLS (Transport Layer Security) and HTTPS (Hypertext Transfer Protocol Secure) [101]. Moreover, key management features offered by cloud-native encryption systems enable businesses to securely create, save, and rotate encryption keys. In order to guarantee adherence to security guidelines and legal requirements for key management, this involves overseeing the key lifecycle, access controls, and auditing capabilities.

Cloud-native encryption solutions facilitate the seamless encryption and decryption of data across distributed systems by providing integration with cloud-native services and applications. To encrypt data at the application level and apply encryption policies uniformly across various components and contexts, entails integrating databases, object storage, and messaging services. Ultimately, by offering an extra layer for encrypting data in transit and at rest and guaranteeing the security, integrity, and accessibility of highly sensitive data across distributed systems, cloud-native encryption solutions play a crucial part in protecting cloud-native applications.

#### 4.1.10. Runtime Protection Platforms

Runtime security technologies, like Sysdig Secure and Aqua Security, monitor, identify, and react in real time to security risks due to secure cloud-native applications and infrastructure [102]. These solutions enable firms to protect against sophisticated assaults that target cloud-native environments by offering additional features for workload protection, container security, and runtime visibility. Container runtime security, which monitors file integrity, network interactions, and container activities to identify unusual activity and possible security issues, is one of the main components of runtime protection solutions. This enables operators to react quickly and reduce security risks by identifying suspicious activity, privilege escalation, and unauthorized access within containers.

Runtime protection platforms support workload protection, which involves protecting cloud-native workloads and apps operating on container orchestration platforms like Docker and Kubernetes, along with container runtime security. This entails keeping an eye on workload patterns, looking for signs of compromise, and implementing security regulations to stop illegal access and data leakage. Runtime security platforms also offer visibility and analytics tools, including reports, dashboards, and warnings, which let businesses learn more about their runtime precautions and spot new risks. In cloud-native environments, this helps enterprises keep ahead of evolving security threats by enabling proactive threat hunting, incident response, and forensic analysis. In general, runtime protection technologies are essential for protecting cloud-native applications because they offer constant runtime monitoring, detection, and response capabilities, guaranteeing the availability, integrity, and confidentiality of workloads and data in dynamic and distributed environments.

#### 4.1.11. Cloud-Native Endpoint Security

In cloud-native environments, endpoints, devices, and apps are protected against sophisticated threats, malware, and cyberattacks by cloud-native endpoint security solutions like next-generation antivirus (NGAV) and CrowdStrike Falcon [103]. These solutions help enterprises protect their cloud-native applications and infrastructure by offering complete endpoint protection capabilities including antivirus, endpoint detection and response (EDR), and threat hunting. EDR, which entails tracking endpoint activity, spotting suspicious activity, and instantly reacting to security problems, is one of the main components of cloud-native endpoint protection. To stop more harm to endpoints and data entails seeing signs of compromise, looking into security incidents, and managing threats.

Cloud-native endpoint security solutions allow threat hunting as well as end-to-end data recovery EDR. Threat hunting is the proactive search for indications of malicious activity and potential security concerns in cloud-native environments. This entails performing forensic investigations, examining endpoint telemetry data, and spotting any hidden dangers that could be achieved through conventional security measures. Moreover, cloud-native endpoint security solutions facilitate the deployment and oversight of endpoint protection agents across distributed environments via integration with cloud-native platforms and services. This ensures thorough security for all endpoints in cloud-native environments and includes support for serverless applications, virtualized infrastructure, and containerized processes.

Advanced preventive features like NGAV, machine learning-based threat detection, and behavioral analysis are also provided by cloud-native endpoint security systems. Preventing malware infections, file-less assaults, and zero-day vulnerabilities helps businesses reduce the risk of security lapses and data losses in cloud-native environments. In general, cloud-native endpoint security solutions are essential for protecting cloud-native infrastructure and applications because they offer proactive threat hunting, real-time threat detection, and advanced endpoint protection features that ensure endpoint security and resilience in dynamic and dispersed environments.

#### 4.1.12. Cloud-Native Zero-Trust Security

Securing network traffic, communications, and connection inside cloud-native environments is the main goal of cloud-native zero-trust platform solutions like Palo Alto Networks Prisma Access and Cisco Umbrella. By offering sophisticated network segmentation, traffic inspection, and threat prevention features, these solutions assist organizations in defending their cloud-native infrastructure against online threats and assaults. Network segmentation, which divides cloud-native environments into separate security zones or segments to stop threats from moving laterally and lessen the effect of security breaches, is one of the essential components of cloud-native network security [104]. Maintaining security limits and regulating traffic flow between segments entails putting in place virtual firewalls, access restrictions, and micro-segmentation policies.

Advanced threat prevention features like encrypted traffic inspection, DNS security, and zero-trust networking are also available with cloud-native network security solutions. Preventing data exfiltration, command-and-control (C2) communication, and other network-based assaults aids businesses in lowering the possibility of security lapses and data loss in cloud-native environments. Ultimately, these solutions offer advanced network protection capabilities, traffic inspection, and threat prevention, all of which ensure the reliability, accessibility, and privacy of network resources within the dynamic and distributed environments for which cloud-native network security solutions play a critical role in protecting cloud-native applications and infrastructure.

#### 4.1.13. Cloud-Native Security Information and Event Management (SIEM)

Cloud-native environments can benefit from the centralized monitoring, analysis, and reporting of security events through the use of SIEM solutions like IBM QRadar, LogRhythm, and Splunk Enterprise Security [105]. To quickly identify and address security events, these systems gather, correlate, and analyze log data from a variety of sources, such as cloud services, apps, and infrastructure parts. Log collection and aggregation, which entails feeding log data from several sources, including cloud platforms, network devices, and security appliances, into a centralized repository for analysis, is one of the main characteristics of cloud-native SIEM solutions. Making the correlation and analysis of security incidents easier involves processing log messages, extracting pertinent information, and standardizing data formats.

Cloud-native SIEM systems provide real-time event correlation and analysis, entailing connecting security events, spotting patterns, and identifying possible security concerns, along with log collection and aggregation. Recent research has extended this concept by incorporating federated learning and blockchain into intrusion detection architectures, enabling distributed and tamper-proof detection mechanisms for zero-trust environments [106]. This entails analyzing log data using guidelines, heuristics, and statistical algorithms to produce alerts for anomalies suspicious activity and indications of compromise (IOCs). Moreover, incident response and workflow automation features offered by cloud-native SIEM solutions enable enterprises to expedite security incident management, investigation, and remediation procedures. This entails coordinating response methods such as severing hacked assets, blocking malicious IP addresses, and reporting issues to security teams for additional investigation and handling.

Furthermore, cloud-native SIEM solutions facilitate the easy deployment and management of security measures in dynamic and distributed environments by providing integration with cloud-native platforms and services. Support for serverless apps, virtualized infrastructure, and containerized tasks are all included in this, ensuring comprehensive asset protection and visibility in cloud-native environments. All things considered, cloud-native SIEM solutions are essential for protecting cloud-native infrastructure and applications because they offer centralized security event monitoring, analysis, and response capabilities. This aids in the efficient detection, investigation, and handling of security incidents by businesses operating in dynamic and dispersed environments.

#### 4.1.14. Cloud-Native Threat Intelligence Platforms

ThreatConnect, Anomali, and Recorded Future are a few examples of cloud-native threat intelligence platforms that assist enterprises in proactively preventing cyberattacks. These platforms gather, examine, and distribute threat intelligence from a variety of sources, including open-source feeds, for-profit suppliers, and internal security telemetry data. Threat data aggregation and enrichment, which entail gathering and compiling threat intelligence feeds from many sources, such as vulnerability databases, malware repositories, and security research reports, are among the primary characteristics of cloud-native threat intelligence platforms [107]. The provision of thorough insights into new threats and attack vectors involves standardizing data formats, adding contextual information to threat indicators, and tying relevant events together.

Threat analysis and prioritization, which entail evaluating threat intelligence data, identifying pertinent threats, and ranking them according to risk level, impact, and likelihood of exploitation, are supported by cloud-native threat intelligence platforms in conjunction with threat data aggregation and enrichment. This entails using machine learning algorithms, threat grouping strategies, and risk assessment models to prioritize actionable intelligence and concentrate resources on countering high-priority threats. Additionally, threat intelligence solutions that are cloud-native offer integration with security products and platforms, making it possible for threat intelligence to be shared and disseminated across distributed environments with ease. This involves integrating security telemetry data with contextual threat intelligence and improving threat detection and response capabilities through interaction with SIEM solutions, EDR technologies, and threat detection platforms [108].

Furthermore, cloud-native threat intelligence tools help security teams work together and share information more easily, which makes it possible to react quickly to new threats and vulnerabilities. These tools assist businesses in staying ahead of changing cyber threats and adjusting their security posture by providing ongoing monitoring and analysis. Cloud-native threat intelligence techniques offer predictive insights into future threats and trends by utilizing machine learning and advanced analytics. The investigation and mitigation of security issues can be expedited through integration with incident response platforms. All things considered, Table 3 demonstrates the cloud-native threat intelligence tools that enable enterprises to fortify their security stance, reduce risks, and successfully defend their cloud-native environments against cyberattacks.

Finally, a growing array of tools and methods for protecting cloud-native applications is now accessible, reflecting how dynamic cybersecurity is in today’s digital environment. Organizations may guarantee the integrity of their cloud-native deployments, reduce risks, and improve their security posture by utilizing these new developments as demonstrated by Table 3. Organizations need to continue to be watchful and proactive in implementing the newest technologies and best practices for protecting their cloud-native apps as cyber threats and attack vectors advance in sophistication. The industry can jointly increase the resilience of cloud-native settings and clear the path for a more secure digital future by collaborating and ensuring constant innovation.

### 4.2. Comparative Analysis of Cloud-Native Security Techniques

While each cloud-native security technique serves a distinct function, their adoption often hinges on trade-offs between performance, scalability, and the ease of integration. A comparative overview is essential for organizations aiming to tailor their security stack based on architectural requirements and operational priorities. For instance, service mesh platforms such as Istio or Linkerd offer excellent scalability and observability, but their sidecar proxy model introduces moderate performance overhead, making them less ideal for latency-sensitive workloads. On the other hand, runtime protection platforms like Sysdig Secure provide lightweight, real-time protection with high compatibility across platforms, making them easy to integrate within container orchestration environments.

IAM solutions such as AWS IAM and Google Cloud IAM provide seamless integration with cloud providers and low performance impact but may lack fine-grained control in multi-cloud environments compared to service mesh access policies. AI-driven threat detection and SIEM platforms deliver unmatched visibility and real-time analytics at scale but typically require substantial compute resources and specialized integration with existing telemetry pipelines. Furthermore, immutable infrastructure and DevSecOps pipelines provide exceptional consistency and automation, but their initial setup demands careful planning and high integration effort with CI/CD tools. Container image scanning tools are relatively lightweight and easy to integrate, especially in early development phases, but do not address runtime or network-layer threats, which runtime protection and service mesh technologies cover more effectively.

This variation in strengths across tools highlights a deeper contrast with traditional monolithic security models such as centralized firewalls, host-based antivirus, and perimeter-based access controls which were designed for static infrastructures rather than dynamic cloud-native ecosystems. Traditional tools are often static and perimeter-focused, which limits their effectiveness in dynamic, containerized, and decentralized environments. For example, while legacy antivirus solutions offer protection at the device level, cloud-native endpoint security platforms enable continuous monitoring across ephemeral workloads, containerized services, and virtualized infrastructure. Similarly, DevSecOps pipelines and runtime protection platforms provide automated, integrated defense mechanisms that align directly with CI/CD workflows and microservices deployment capabilities that traditional approaches lack. These improvements reflect a paradigm shift from reactive to proactive, infrastructure-integrated security models designed for the agility and scale of modern cloud-native applications. Hence, this comparative insight emphasizes that no single solution is universally optimal; rather, a strategic combination tailored to the cloud-native environment is essential to achieving a robust, scalable, and efficient security posture.

Thus, Table 4 provides a comparative overview of key cloud-native security techniques, evaluating each method based on its performance impact, scalability, the ease of integration, and associated trade-offs to support strategic selection in diverse deployment environments. While comparative analysis highlights functional and operational trade-offs, it is equally important to understand the theoretical rationale for why these techniques are inherently suitable for cloud-native architectures. The following subsection offers such justification.

### 4.3. Theoretical Justification of Security Techniques in Cloud-Native Environments

The effectiveness of specific security techniques in cloud-native environments is inherently tied to the unique architectural and operational characteristics of these systems. Traditional perimeter-based security models fall short in dynamic, distributed microservice architectures where components are loosely coupled, and workloads are ephemeral. For example, runtime protection platforms are particularly effective because cloud-native applications often operate in containers that instantiate and terminate rapidly, making static scanning insufficient. Real-time monitoring and response are therefore essential to address threats that emerge during execution.

Service mesh technologies offer theoretical advantages in cloud-native systems due to their ability to abstract network policies from application logic. Given the fluid nature of service-to-service communication across microservices, having a dedicated control layer for mutual TLS, circuit breaking, and observability improves both security and reliability without modifying the application code. Similarly, immutable infrastructure principles are well aligned with cloud-native ideals of declarative configuration and reproducibility. By treating infrastructure as disposable and version-controlled, organizations reduce drift, ensure consistent security baselines, and enable rapid recovery from breaches.

The DevSecOps model is not only the best practice but a necessity in cloud-native development, where continuous integration and deployment pipelines are integral. Embedding security checks directly into CI/CD ensures that security evolves with the application, rather than lagging behind. Furthermore, cloud-native IAM and zero-trust networking address the absence of a fixed network boundary, securing access based on identity and context rather than location—crucial for multi-cloud and hybrid deployments.

These theoretical alignments between technique and architecture underscore the need for security models that are adaptive, decentralized, and automated, thereby making them not only effective but foundational in securing cloud-native applications.

## 5. Case Scenario: Ensuring Security in Cloud-Native Applications

**Figure 4:**
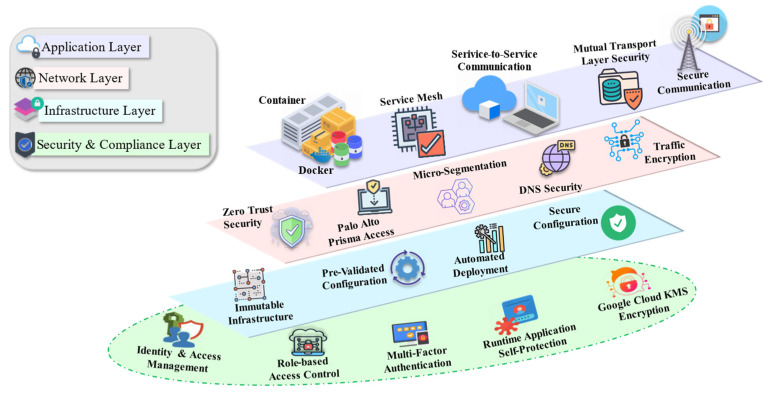
Multi-layered security architecture for cloud-native applications, illustrating protective measures applied across applications, network, infrastructure, and compliance layers.

In modern cloud-native environments, applications are often composed of multiple microservices running on distributed infrastructures. This architecture offers significant advantages in terms of scalability and agility, but it also introduces complex security challenges. Each microservice, container, and communication channel must be secured to protect sensitive data, maintain service integrity, and prevent unauthorized access. As shown in Figure 4, the security architecture integrates multiple layers, with the security and compliance layer serving as an overarching layer that secures all other components. This case scenario illustrates how cloud-native security solutions are applied across different layers such as application, network, infrastructure, and security to ensure robust protection in a cloud-native architecture.

### 5.1. Application Layer

The application layer serves as the foundation of cloud-native architecture, hosting multiple microservices that are responsible for key business functions, such as user authentication, data processing, and transaction management. These microservices run in isolated containers to ensure security and scalability. However, this layer is vulnerable to unauthorized access, data interception, and man-in-the-middle (MITM) attacks, particularly when services communicate across different environments. To mitigate these risks, service mesh technologies, such as Istio, are implemented to manage inter-service communications. Mutual Transport Layer Security is employed within the service mesh to encrypt all communication and ensure that both the client and server authenticate each other’s identities before establishing a connection. This encryption mechanism significantly reduces the risk of unauthorized access or data leaks. Additionally, mTLS offers robust defense against MITM attacks in which a hacker could intercept and modify data flowing between services. These security controls reduce the likelihood of data breaches by maintaining the confidentiality and security of microservices communication.

### 5.2. Network Layer

Once the application layer is secured, attention shifts to the network layer, which is responsible for managing communication between microservices across different network nodes. Securing internal communications within this layer is critical to prevent the lateral movement of attackers across the network if any part of the system is compromised. Without proper security measures, threats could spread quickly across multiple services, leading to data breaches or system outages. To address this, the organization adopts a zero-trust security model, which follows the principle of “never trust, always verify”. Tools like Palo Alto Prisma Access are deployed to continuously monitor and segment network traffic, ensuring that every connection within the network is authenticated and authorized. Micro-segmentation is applied to divide the network into smaller, isolated security zones. This approach prevents attackers from moving freely between services, limiting the impact of a breach to the compromised zone. Additionally, DNS Security and Traffic Encryption are implemented to monitor network traffic and protect it from external and internal attacks. These measures ensure that any potential breaches are contained, and that sensitive data remain secure as they travel throughout the network.

### 5.3. Infrastructure Layer

The infrastructure layer, which supports the dynamic cloud-native environment, is highly susceptible to misconfigurations, vulnerabilities, and human errors due to the frequent updates and changes in containerized applications. Maintaining consistency and security across infrastructure deployments is essential to ensure that the system remains reliable and protected from attacks. To overcome these challenges, immutable infrastructure practices are adopted. Using Terraform for infrastructure-as-code, the organization ensures that each deployment follows predefined templates, preventing configuration drift and ensuring security across all environments. In cases where vulnerabilities or misconfigurations are detected, the infrastructure is automatically replaced with a secure, known-good state. This approach minimizes downtime, reduces the risk of configuration-related vulnerabilities, and ensures that the environment always remains secure and operational. Additionally, Automated Deployment processes validate all changes before deployment, reducing the risk of human error and enhancing the overall security of the infrastructure.

### 5.4. Security and Compliance Layer

The security and compliance layer plays a critical role in safeguarding all the other layers such as application, network, and infrastructure, by applying overarching security measures that ensure the entire system remains protected and compliant. Instead of functioning as a discrete layer, it wraps around and integrates with each of the other layers, enforcing security protocols across the entire architecture. At the application layer, the security and compliance layer ensures that microservices adhere to Role-Based Access Control and that access is tightly controlled through IAM systems, such as AWS IAM. It also implements multi-factor authentication to provide an additional layer of security for critical operations, such as managing financial data or handling customer information. In the network layer, the security and compliance layer continues to enforce zero-trust security principles, ensuring that even within segmented and isolated zones, every connection is authenticated and authorized. Traffic Encryption and DNS Security are implemented as overarching measures, protecting network traffic from unauthorized interception and ensuring data confidentiality across all communication channels.

At the infrastructure layer, the security and compliance layer governs the use of immutable infrastructure by enforcing secure deployment practices and ensuring that Automated Deployment processes are compliant with organizational security policies. By monitoring changes in real time, it reduces the chances of misconfigurations and ensures that any vulnerabilities are quickly addressed. Moreover, runtime application self-protection solutions, like Sysdig Secure, are integrated across all layers to monitor real-time threats such as privilege escalation or code injection. RASP continuously detects suspicious behavior in applications, containers, and infrastructure, taking immediate action to mitigate risks before they escalate. Cloud-native encryption solutions, such as Google Cloud Key Management Service, further enhance security by encrypting data, both in transit and at rest, ensuring that sensitive information remains secure even if intercepted.

Thus, this demonstrates the importance of applying security measures across multiple layers in a cloud-native architecture. By positioning the security and compliance layer as an overarching layer, security protocols are seamlessly applied across the application, network, and infrastructure layers, ensuring a unified security posture. Technologies like service mesh, zero-trust security, immutable infrastructure, and RASP offer robust defense mechanisms, protecting the entire architecture from evolving cyber threats. These practices ensure that data and applications remain secure, resilient, and compliant, even in dynamic cloud-native environments.

Beyond ensuring multi-layered protection, this case scenario also emphasizes the distinct operational advantages of individual cloud-native security techniques and how they complement one another. For example, service mesh provides encrypted inter-service communication, RASP enables real-time threat mitigation during execution, and IAM enforces strict access controls across services. These tools address different threat vectors, showcasing their complementary nature. Furthermore, when contrasted with traditional monolithic security models such as perimeter-based firewalls or host-level antivirus, the proposed architecture introduces automation, continuous monitoring, micro-segmentation, and scalability. This layered, integrated model shifts security from reactive to proactive, aligning with the fluidity and complexity of modern cloud-native infrastructures. As such, it represents a marked advancement over legacy systems in both responsiveness and resilience.

## 6. Challenges and Solutions

Several difficulties arise while developing secure cloud-native applications because of the dynamic nature of cloud environments and the special qualities of containerized architectures. The security of handling and storing sensitive data in cloud-native apps is one of the main issues. The effective protection of user information and personal data is essential for maintaining compliance with privacy regulations and reducing legal, financial, and reputational risks. Furthermore, even while scalability has many advantages, there are security risks that should be carefully considered.

### 6.1. Dynamic Nature of Cloud-Native Environments

The dynamic architecture of cloud-native environments, which is typified by microservices and containers expanding quickly in response to demand, makes it difficult to keep things stable and safe. This could result in runtime vulnerabilities because traditional security solutions made for static systems might not be able to keep up with the dynamic changes. Furthermore, programs operating in containers may be subject to security threats resulting from untrusted host OS vulnerabilities, making container runtime security an extremely important consideration.

### 6.2. Malware Detection

The detection of complex malware intended for containerized apps may be difficult using traditional methods in cloud-native systems, posing another hurdle. Cybercriminals take advantage of this by using sophisticated software that eludes standard detection techniques, including malware designed to mine cryptocurrencies. Furthermore, the integrity of the whole cloud-native system is seriously threatened by host OS kernel vulnerabilities, which may result in illegal access, data breaches, or other security issues.

### 6.3. Unauthorized Access and Data Breaches

To stop unwanted access and data breaches, microservices must have strong access control mechanisms and secure inter-container connections. The security and integrity of the system can be risked by attackers who can intercept or alter communication across microservices in the absence of sufficient security protections. Moreover, applications’ responsiveness and availability might be interfered with by distributed denial-of-service (DDoS) assaults directed toward cloud containers, leading to service outages and downtime.

### 6.4. Decentralized Access Control

Microservice architectures with decentralized access control are more complex because centralized systems can have single points of failure, which can allow unauthorized access to spread widely among microservices in the event of a breach. Finally, it can be difficult to create realistic testbeds to assess security systems in microservices-based cloud-native environments, which can lead to security measures being overlooked because of insufficient testing conditions. All things considered, overcoming these obstacles calls for a comprehensive strategy that incorporates strong security controls made specifically for cloud-native applications as highlighted by Table 5.

In consequence, the creation of more sophisticated security solutions designed especially for dynamic cloud-native systems is one encouraging path. Modern technologies like artificial intelligence and machine learning should be included in these solutions to enable real-time threat detection and response. Consequently, improved container runtime security methods that offer better isolation between containers and the underlying host OS are also becoming increasingly necessary. Lessening the effect of host OS vulnerabilities on containerized apps may entail sandboxing and the deployment of sophisticated runtime protections. The adoption of increasingly advanced detection methods that can recognize sophisticated malware created especially for containerized apps holds the key to the future of malware detection in cloud-native environments. Successfully identifying and reducing risks may entail the use of behavior-based analysis and anomaly detection algorithms. Moreover, enhancing access control methods for inter-container interactions and microservices is another essential component. In the future, it might be possible to create decentralized access control systems that disperse access control policies over several nodes, lowering the possibility of unwanted access and single points of failure.

Furthermore, it will be crucial to take preventative action to lessen the impact that DDoS attacks can have on cloud containers. This can entail implementing scalable DDoS mitigation systems that can adjust to shifting traffic loads and attack patterns spontaneously. Eventually, testing environments that are more detailed and realistically represent the complexity of cloud-native deployments should be developed. Future work may focus on creating sophisticated testing frameworks and simulation tools that offer a more realistic depiction of cloud-native settings in real life, helping enterprises detect and resolve security flaws more quickly. Overcoming the obstacles encountered by cloud-native applications necessitates a proactive strategy that leads to emerging trends and technological breakthroughs. Over the past decade, cloud-native security has undergone a significant transformation, shifting from traditional perimeter-based defenses to more adaptive and decentralized protection mechanisms. Early approaches such as static firewalls and monolithic access controls proved inadequate in handling the distributed, dynamic nature of microservices and containers. This drove the adoption of DevSecOps pipelines, runtime security, and infrastructure-as-code auditing to enable real-time protection within CI/CD workflows. In recent years, the focus has further evolved toward zero-trust architectures, service mesh-based micro-segmentation, AI/ML-powered threat detection, and blockchain-enabled access control. These trends reflect a clear trajectory toward intelligent, context-aware, and scalable security solutions tailored to dynamic cloud-native environments. Thus, the creation of increasingly sophisticated and adaptable security solutions, designed especially for dynamic cloud environments, is where cloud-native application security is headed in the future. This entails making use of state-of-the-art technology, increasing access control methods, enhancing malware detection capabilities, enhancing container runtime security, reducing the impact of DDoS attacks, and improving testing and assessment methodologies.

## 7. Conclusions

The development, deployment, and management of applications have completely changed with the advent of cloud-native architecture. Cloud-native environments present certain security and trust-related challenges that must be addressed to ensure the availability, integrity, and privacy of data and applications. This study has examined current developments in cloud-native application security tools and methodologies, such as runtime protection solutions, cloud-native threat intelligence platforms, and DevSecOps pipelines. Through comparative architectural analysis and a scenario-based demonstration, the paper highlighted how these solutions can enforce regulatory compliance, proactively mitigate threats, and strengthen overall trust in dynamic environments. However, there are several difficulties in developing secure cloud-native applications, including the dynamic and distributed nature of cloud-native environments and the complexity of protecting communications across containers. To tackle these obstacles, this study has provided a case study that shows how security control can be applied at multiple layers, including application, network, infrastructure, and security and compliance. This layered model not only illustrates operational implementation but also emphasizes how each technique aligns with cloud-native design principles. Looking ahead, future research will likely focus on developing even more sophisticated security solutions that leverage advancements in machine learning and artificial intelligence. These technologies are expected to revolutionize container runtime security, enhance privacy-aware access-control mechanisms, and strengthen trust-centric security models to mitigate the effects of DDoS attacks. Additionally, the integration of blockchain technology could provide new ways to manage and verify identities in cloud-native environments, offering improved transparency and security. The progression towards more integrated and intelligent security solutions will be critical in addressing the complexities of protecting cloud-native infrastructures, grounding conclusions in the analyzed trade-offs and architectural fit rather than empirical data. By embracing privacy-enhancing, multi-layered security models and investing in forward-looking innovations, organizations can confidently navigate the complexities of cloud-native environments, ensuring the trustworthiness, security, and adaptability of their applications in today’s rapidly evolving digital landscape.

## Figures and Tables

**Table 1:** Issues and implementations of latest review papers for enhancing cloud-native security.

Authors	Focus Area	Issues	Description	Tools and Techniques
[31]	The dynamic nature of cloud-native security	Addressing swift alterations in containerized architectures	Examined how dynamic cloud-native settings affect security, with a focus on adaptable security measures.	Modular threat modeling and adaptable security measures
[33]	Microservice architecture’s effects on security	Reducing risks related to decentralized patterns and illegal access	Examined how microservice designs in cloud-native contexts affect security.	Decentralized access control systems with robust authentication techniques
[35,36]	DevSecOps pipelines	Fostering a development strategy that prioritizes security	Explored how DevSecOps pipelines may automate security testing along the software development lifecycle.	Vulnerability scanning and automated security testing
[38]	Leveraging threat intelligence	Overcoming obstacles in the proactive defense against cyberattacks	Investigated integrating cloud-native threat intelligence systems with pipelines for DevSecOps.	Threat data collection and evaluation
[40]	Runtime security for containers	Preventing runtime errors in programs that are containerized	Examined the difficulties in protecting containerized applications from runtime vulnerabilities.	Isolating containers and reducing host OS vulnerabilities
[42]	Enhanced runtime security	Securing cloud-native environments by implementing policies into place	Investigated the application of sandboxing and sophisticated runtime security measures in cloud-native environments.	Techniques for sandboxing and runtime protection
[46]	Automation of compliance	Ensuring adherence to the CCPA, GDPR, and other privacy laws	Examined the difficulties in ensuring that cloud-native applications comply with privacy laws and regulations.	Access control methods and data encryption
[54]	AI-based security solutions	Overcoming obstacles in cloud-native environments related to cyber threat detection and mitigation	Explored the possibilities for identifying and reducing risks in cloud-native environments using AI-driven security solutions.	Security telemetry analysis and machine learning techniques
[55]	AI-based security solutions	Overcoming obstacles to effectively utilizing AI for threat detection and mitigation	Explored the possibilities of AI-driven security solutions, with an emphasis on threat identification and mitigation, for cloud-native IoT environments.	Threat detection and mitigation using machine learning techniques
[56]	Blockchain-driven security	Addressing single points of failure and preventing illegal access	A decentralized access control mechanism that uses blockchain technology in microservice architectures is proposed.	Blockchain-driven system for access control
[61]	Observance of legal requirements	Ensuring compliance with best practices and regulatory standards	Addressed how to match established frameworks, such as the NIST Cybersecurity Framework, with cloud-native security concepts.	Combining compliance requirements with security controls
[63,64]	DevSecOps procedures and culture	Addressing issues with ongoing security maintenance across the software development lifecycle	Discovered how to include security procedures in DevOps processes while valuing teamwork.	Collaboration between teams and integration of DevSecOps
[65]	Serverless architecture security considerations	Overcoming obstacles of putting strong authentication and data security measures in place in serverless applications	Examined serverless architecture security issues, focusing on data protection and authentication techniques.	Data protection and authentication procedures
[66]	Edge computing security issues	Addressing issues with data transmission and edge device security in decentralized infrastructures	Examined edge computing infrastructures’ best practices and security issues.	Security of edge devices, data transfer, and application deployment
[67]	Edge computing security concerns	Keeping decentralized edge infrastructures secure	Examined edge computing environments’ security problems and recommendations, with a focus on protecting edge devices and data transit.	Security of edge devices and data transfer

**Table 2:** Comparative analysis of cloud-native applications and their impact.

Applications	Objective	Technologies Used	Benefits
Web applications	Provide high-availability, accessible, and scalable applications via web browsers	High-availability configurations, cloud infrastructure deployment, and dynamic scaling	Improved user experience, global accessibility, and cost efficiency.
Microservice-based applications	Allow application components to be developed, deployed, and scaled independently	APIs for communication, decentralized architecture, and small, independent services	Enhanced fault isolation, faster time-to-market, and improved agility.
Containerized applications	Ensure that deployment environments are consistent and isolated	Encapsulating dependencies, Kubernetes for orchestration, and Docker for containerization	Ease of deployment, enhanced resource utilization, and consistent behavior across environments.
Serverless applications	Abstract server administration to enable business logic-focused development	Event-driven execution, Google Cloud Functions, AWS Lambda, and Azure Functions	Faster development cycles, costly effective, automatic scaling.
IoT (Internet of Things) applications	Gather, handle, and evaluate data from linked devices.	Sensor integration, cloud-based analytics frameworks, and real-time data processing	Enhanced operational efficiency, improved decision-making, and real-time insights.
Big data and analytics applications	Process and examine huge datasets to find insights	Cloud storage possibilities, Google BigQuery, Apache Spark, and Hadoop	Advances in scientific research, corporate intelligence, and informed decision-making.
Real-time messaging and collaboration applications	Facilitate immediate collaboration and communication.	Low-latency networks, cloud-native communications protocols, and real-time data synchronization	Improved user interaction, enhanced remote collaboration, and seamless communication.
Blockchain-based applications	Enhance security and transparency while hosting decentralized applications	Decentralized ledgers, cloud-hosted nodes, and blockchain frameworks	Improved trust, secure transactions, and decentralized control.
AI and machine learning applications	Enable large-scale data processing and advanced computations for AI models	Cloud-based platforms with GPUs and TPUs, machine learning frameworks, and scalable computing resources	Rapid creation of AI models, scalable computing, and customized user interfaces.
Monitoring and observability applications	Assure cloud-native applications reliability and efficiency	Grafana for visualization, Elasticsearch for logging, and Prometheus for monitoring	Enhanced system health, proactive issue resolution, and optimized performance.
Healthcare and telemedicine applications	Improving patient care and enabling remote health services	Secure data storage, real-time communication tools, and cloud-based EHR systems	Improved patient care, compliance with health laws, and remote diagnosis.
API management platforms	Make API design, administration, and security easier	Kong, AWS API Gateway, Apigee, and access control methods	Speedy application development, secure data transfer, and improved API governance.

**Table 3:** Recent tools and techniques for securing cloud-native applications.

Tool and Technique	Purpose	Key Features	Implementation Method	Impact
Service mesh	Handle service-to-service communication	Traffic management, observability, mTLS encryption.	Istio, Linkerd, Consul Linkerd, Istio, and Consul	Improved security, resilience, and observability
Runtime application self-protection	Monitor real-time application security	Anomaly detection, vulnerability scanning, behavioral analysis.	Trivy Aqua Security, Sysdig Secure	Proactively identify threats and response
Serverless security	Protected serverless environments	Runtime monitoring, access control, and function isolation.	Azure Functions Proxies, AWS Lambda Layers	Improved security, minimized risk of unauthorized access
Security and compliance management	Centralized compliance handling and security	Threat detection, compliance reporting, and network segmentation.	Palo Alto Networks Prisma Cloud, Aqua CSPM	Enhanced security posture, regulatory compliance
Immutable infrastructure	Assure consistency and protected infrastructure	Automated provisioning, configuration management, IaC templates.	AWS CloudFormation, HashiCorp Terraform	Lessened attack surface, robust rollback, and recovery
DevSecOps pipelines	Secure the CI/CD pipeline by integration.	Compliance checks, automated security testing, vulnerability scanning.	SonarQube, GitLab Secure, and Snyk	Automating compliance and deploying secure code
Cloud-native IAM	Handle identities and access controls	Identity federation, MFA, and granular access control	Google Cloud IAM and AWS IAM	Diminished risk of unauthorized access, improved security
Container image scanning	Determine which container images are vulnerable.	Integration with CI/CD, compliance scanning, and vulnerability detection.	Anchore Engine, Clair, Docker Security Scanning	Reduced risk of deploying insecure containers
Cloud-native encryption	Secure data both in transit and at rest.	Key management, encryption techniques, and cloud service integration	HashiCorp Vault, Google Cloud KMS, and AWS KMS	Secure sensitive data, and assure data confidentiality
Runtime protection platforms	Monitor real-time security and response	Workload protection, visibility, container runtime security, and analytics	Sysdig Secure and Aqua Security	Continuous incident response and threat monitoring
Cloud-native endpoint security	Secure endpoints and devices	Threat hunting, advanced prevention capabilities, and EDR	Carbon Black Cloud, CrowdStrike Falcon	Improved endpoint security, real-time threat detection
Cloud-native zero-trust Security	Protect network traffic and communications	Traffic inspection, threat prevention, network segmentation,	Cisco Umbrella and Palo Alto Networks Prisma Access	Limited network access and prevented data exfiltration
Cloud-native SIEM	Centralized security incident tracking and analysis	Automation of incident response, log aggregation, and real-time event correlation.	IBM QRadar, Splunk Enterprise Security, and LogRhythm	Handling comprehensive security incident
Cloud-native threat intelligence platforms	Proactively protect against cyber attacks	Collaboration, threat data aggregation, threat analysis, and prioritization.	Recorded Future, Anomali, and ThreatConnect	Improved security posture and proactive threat reduction

**Table 4:** Comparative analysis of key cloud-native security techniques based on performance, scalability, and integration trade-offs.

Techniques	Performance Impact	Scalability	Ease of Integration	Trade-Offs
Service mesh (Istio, Linkerd)	Medium (proxy overhead)	High	Moderate	Great for network control but adds latency
Runtime protection (Sysdig)	Low	High	High	Lightweight, strong at runtime, easy to integrate
IAM (AWS IAM, Google IAM)	Low	Medium	High	Seamless for cloud-native, but limited across clouds
AI/threat detection (SIEM)	High	High	Moderate	Requires computing power and telemetry integration
DevSecOps pipelines	Low–medium	High	Moderate–high	High automation but setup complexity
Image scanning tools	Low	Medium	High	Best in CI/CD; not effective for runtime threats

**Table 5:** Privacy concerns and solutions in securing cloud-native applications.

Concerns	Restrictions and Limitations	Challenges and Issues	Solutions
Security issues	Because cloud-native settings are dynamic, it can be difficult to maintain security and stability.	Handling sensitive data, adhering to privacy rules, and scalability issues are examples of security hazards.	Creation of cutting-edge security solutions that make use of AI/ML, improved malware detection methods, and strengthened container runtime security.
Malware identification	Sophisticated malware created for containerized applications may be undetectable to traditional detection methods.	Cybercriminals can harm the entire system by taking advantage of flaws in the host OS kernels.	Deployment of advanced detection methods for successful threat mitigation, such as anomaly detection algorithms and behavior-based analysis.
Access management	Unauthorized access and data breaches can result from insufficient access control measures.	To avoid security breaches, inter-container communications must be secured.	Enhancing access control measures and creating decentralized systems to lower the possibility of unwanted access and single points of failure.
Examining conditions	It is difficult to build realistic testbeds for assessing security systems in environments based on microservices.	Errors in security measures could be caused by improper testing conditions.	Creation of sophisticated testing frameworks and modeling tools to precisely replicate cloud-native settings in the real world.
Scalability consequences	Although scalability has advantages, it also brings security risks that should be carefully considered.	Runtime vulnerabilities could arise from cloud-native environments’ dynamic nature.	To lessen the effect of DDoS assaults on cloud containers, scalable DDoS mitigation technologies, improved container runtime security, and preventive measures should be implemented.

## Data Availability

Data sharing is not applicable.

## References

[1] Mustyala A. (2023). Migrating Legacy Systems to Cloud-Native Architectures for Enhanced Fraud Detection in Fintech. EPH-Int. J. Sci. Eng..

[2] Atieh A.T. (2021). The next Generation Cloud Technologies: A Review on Distributed Cloud, Fog and Edge Computing and Their Opportunities and Challenges. Res. Rev. Sci. Technol..

[3] Russo E., Longo G., Guerar M., Merlo A. (2023). Cloud-Native Application Security Training and Testing with Cyber Ranges. Lect. Notes Netw. Syst..

[4] Alka T.A., Sreenivasan A., Suresh M. (2025). Entrepreneurial Strategies for Sustainable Growth: A Deep Dive into Cloud-Native Technology and Its Applications. Futur. Bus. J..

[5] Surianarayanan C., Chelliah P.R. (2023). Demystifying the Cloud-Native Computing Paradigm. International Conference on Ubiquitous Computing and Ambient Intelligence.

[6] Chippagiri S., Ravula P. (2021). Cloud-Native Development: Review of Best Practices and Frameworks for Scalable and Resilient Web Applications. Int. J. New Media Studie.

[7] Vaño R., Lacalle I., Sowiński P., S-Julián R., Palau C.E. (2023). Cloud-Native Workload Orchestration at the Edge: A Deployment Review and Future Directions. Sensors.

[8] Perducat C., Mazur D.C., Mukai W., Sandler S.N., Anthony M.J., Mills J.A. (2023). Evolution and Trends of Cloud on Industrial OT Networks. IEEE Open J. Ind. Appl..

[9] Khalel M.M., Pugazhendhi M.A., Raj G.R. Enhanced Load Balancing in Kubernetes Cluster by Minikube. Proceedings of the 2022 International Conference on Smart Technologies and Systems for Next Generation Computing (ICSTSN).

[10] Shan C., Xia Y., Zhan Y., Zhang J. (2023). KubeAdaptor: A Docking Framework for Workflow Containerization on Kubernetes. Future Gener. Comput. Syst..

[11] Nguyen N.T., Kim Y. A Design of Resource Allocation Structure for Multi-Tenant Services in Kubernetes Cluster. Proceedings of the 2022 27th Asia Pacific Conference on Communications (APCC).

[12] Li W. Algorithm Design for Kubernetes Load Saturation Scheduling in Deep Learning. Proceedings of the 2023 IEEE 6th International Conference on Automation, Electronics and Electrical Engineering (AUTEEE).

[13] Giommi L., Spiga D., Paladino M., Kuznetsov V., Bonacorsi D. (2025). Developments on the “Machine Learning as a Service for High Energy Physics” Framework and Related Cloud Native Solution. IEEE Trans. Cloud Comput..

[14] Roca M., Lee C.B., Pertiwi A.P., Blume A., Caballero I., Navarro G., Traganos D., Lee B. (2024). Subtidal Seagrass and Blue Carbon Mapping at the Regional Scale: A Cloud-Native Multi-Temporal Earth Observation Approach. GIScience Remote Sens..

[15] Xiong K., Wu Z., Jia X. (2025). DeepContainer: A Deep Learning-Based Framework for Real-Time Anomaly Detection in Cloud-Native Container Environments. J. Adv. Comput. Syst..

[16] Shin D., Kim J., Pawana I.W.A.J., You I. (2025). Enhancing Cloud-Native DevSecOps: A Zero Trust Approach for the Financial Sector. Comput. Stand. Interfaces.

[17] Bharadwaj D., Premananda B.S. Transition of Cloud Computing from Traditional Applications to the Cloud Native Approach. Proceedings of the 2022 IEEE North Karnataka Subsection Flagship International Conference (NKCon).

[18] Manavadariya B., Mangukiya D. (2024). Cloud-Native System Administration: Principles, Practices, Challenges. Pract. Challenges.

[19] Jiao Q., Xu B., Fan Y. Design of Cloud Native Application Architecture Based on Kubernetes. Proceedings of the 2021 IEEE Intl Conf on Dependable, Autonomic and Secure Computing, Intl Conf on Pervasive Intelligence and Computing, Intl Conf on Cloud and Big Data Computing, Intl Conf on Cyber Science and Technology Congress (DASC/PiCom/CBDCom/CyberSciTech).

[20] Shitta-Bey A., Adewole M. (2023). Security Concerns of Cloud Migration and Its Implications on Cloud-Enabled Business Transformation. Ph.D. Thesis.

[21] Ahmadi S. (2024). Zero Trust Architecture in Cloud Networks: Application, Challenges and Future Opportunities. J. Eng. Res. Rep..

[22] Boudi A., Bagaa M., Poyhonen P., Taleb T., Flinck H. (2021). AI-Based Resource Management in beyond 5G Cloud Native Environment. IEEE Netw..

[23] Chen C., Ma W., Gao C., Zhang W., Zeng K., Ye T., Chen Y., Du X. (2025). GaussDB-AISQL: A Composable Cloud-Native SQL System with AI Capabilities. Front. Comput. Sci..

[24] Taleb T., Benzaïd C., Addad R.A., Samdanis K. (2023). AI/ML for beyond 5G Systems: Concepts, Technology Enablers & Solutions. Comput. Networks.

[25] Oyeniran O.C., Modupe O.T., Otitoola A.A., Abiona O.O., Adewusi A.O., Oladapo O.J. (2024). A Comprehensive Review of Leveraging Cloud-Native Technologies for Scalability and Resilience in Software Development. Int. J. Sci. Res. Arch..

[26] Aris A., Levi A., Oz H., Selcuk A., Aris A., Uluagac A.S., Levi A. (2022). A Survey on Ransomware: Evolution, Taxonomy, and Defense Solutions. ACM Comput. Surv..

[27] Malik V., Khanna A., Sharma N. (2024). Trends in Ransomware Attacks: Analysis and Future Predictions. Int. J. Glob. Innov. Solut. (IJGIS).

[28] Lang M., Connolly L.Y., Taylor P., Corner P.J. (2023). The Evolving Menace of Ransomware: A Comparative Analysis of Pre-Pandemic and Mid-Pandemic Attacks. Digit. Threat. Res. Pract..

[29] Bundela R., Dhanda N., Gupta K.K. Identification and Analysis of Security Issues in Cloud Computing. Proceedings of the 2024 2nd International Conference on Disruptive Technologies (ICDT).

[30] Theodoropoulos T., Rosa L., Benzaid C., Gray P., Marin E., Makris A., Cordeiro L., Diego F., Sorokin P., Girolamo M.D. (2023). Security in Cloud-Native Services: A Survey. J. Cybersecur. Priv..

[31] Rahaman S., Islam A., Cerny T., Hutton S. (2023). Static-Analysis-Based Solutions to Security Challenges in Cloud-Native Systems: Systematic Mapping Study. Sensors.

[32] Tatineni S. (2023). AI-Infused Threat Detection and Incident Response in Cloud Security. Int. J. Sci. Research.

[33] Barabanov A., Makrushin D. (2020). Authentication and Authorization in Microservice-Based Systems: Survey of Architecture Patterns. arXiv.

[34] Prakash C. (2024). Zero-Trust Architecture Approach to Secure Microservices for the Healthcare Insurance Industry. Ph.D. Thesis.

[35] Rangnau T., Buijtenen R.V., Fransen F., Turkmen F. Continuous Security Testing: A Case Study on Integrating Dynamic Security Testing Tools in Ci/Cd Pipelines. Proceedings of the 2020 IEEE 24th International Enterprise Distributed Object Computing Conference (EDOC).

[36] Tatineni S. (2023). Compliance and Audit Challenges in DevOps: A Security Perspective. Int. Res. J. Mod. Eng. Technol. Sci..

[37] Continuous F., Klooster T., Turkmen F., Broenink G., Ten Hove R., Bohme M. Continuous Fuzzing: A Study of the Effectiveness and Scalability of Fuzzing in CI/CD Pipelines. Proceedings of the 2023 IEEE/ACM International Workshop on Search-Based and Fuzz Testing (SBFT).

[38] Rangaraju S., Ness S., Dharmalingam R. (2023). Incorporating Ai-Driven Strategies in Devsecops for Robust Cloud Security. Int. J. Innov. Sci. Res. Technol..

[39] Abiona O.O., Oladapo O.J., Modupe O.T., Oyeniran O.C., Adewusi A.O., Komolafe A.M. (2024). The Emergence and Importance of DevSecOps: Integrating and Reviewing Security Practices Within the DevOps Pipeline. World J. Adv. Eng. Technol. Sci..

[40] Ugale S., Potgantwar A. (2023). Container Security in Cloud Environments: A Comprehensive Analysis and Future Directions for DevSecOps. Eng. Proc..

[41] Li Y., Hu H., Liu W., Yang X. (2023). An Optimal Active Defensive Security Framework for the Container-Based Cloud with Deep Reinforcement Learning. Electronics.

[42] Abbadini M., Beretta M., Facchinetti D., Oldani G., Rossi M., Paraboschi S. Lightweight Cloud Application Sandboxing. Proceedings of the 2023 IEEE International Conference on Cloud Computing Technology and Science (CloudCom).

[43] Beauchaine A., Shue C.A. Toward a (Secure) Path of Least Resistance: An Examination of Usability Challenges in Secure Sandbox Systems. Proceedings of the 2023 5th IEEE International Conference on Trust, Privacy and Security in Intelligent Systems and Applications (TPS-ISA).

[44] Shen W., Wu Y., Yang Y., Liu Q., Yang N., Li J., Lu K., Ma J. (2024). Towards Understanding and Defeating Abstract Resource Attacks for Container Platforms. IEEE Trans. Dependable Secur. Comput..

[45] Nakata Y., Suzuki S., Matsubara K. Reducing Attack Surface with Container Transplantation for Lightweight Sandboxing. Proceedings of the 14th ACM SIGOPS Asia-Pacific Workshop on Systems.

[46] Ang’Udi J.J. (2023). Security Challenges in Cloud Computing: A Comprehensive Analysis. World J. Adv. Eng. Technol. Sci..

[47] Igwenagu U., Salami A.A., Arigbabu A.S., Esambe M.C., Oladoyinbo T.O., Olaniyi O.O. (2024). Securing the Digital Frontier: Strategies for Cloud Computing Security, Database Protection, and Comprehensive Penetration Testing. Database Prot. Compr. Penetration Test..

[48] Georgescu T.M. (2021). A Study on How the Pandemic Changed the Cybersecurity Landscape. Inform. Econ..

[49] Yang N., Shen W., Li J., Liu X., Guo X., Ma J. Take over the Whole Cluster: Attacking Kubernetes via Excessive Permissions of Third-Party Applications. Proceedings of the CCS’23: Proceedings of the 2023 ACM SIGSAC Conference on Computer and Communications Security.

[50] Cloud Native Computing Foundation https://www.cncf.io/.

[51] Kamieniarz K., Mazurczyk W. A Comparative Study on the Security of Kubernetes Deployments. Proceedings of the 2024 International Wireless Communications and Mobile Computing (IWCMC).

[52] Zeng Q., Kavousi M., Luo Y., Jin L., Chen Y. (2023). Full-Stack Vulnerability Analysis of the Cloud-Native Platform. Comput. Secur..

[53] Feng M., Zhou J., Tang Y. Enhancing Cloud-Native Security Through EBPF Technology. Proceedings of the 2024 IEEE 11th International Conference on Cyber Security and Cloud Computing (CSCloud).

[54] Arif H., Kumar A., Fahad M., Hussain H.K. (2024). Future Horizons: AI-Enhanced Threat Detection in Cloud Environments: Unveiling Opportunities for Research. Int. J. Multidiscip. Sci. Arts.

[55] Dhayanidhi G. (2022). Research on IoT Threats & Implementation of AI/ML to Address Emerging Cybersecurity Issues in IoT with Cloud Computing. Master’s Thesis.

[56] Xi N., Liu J., Li Y., Qin B. (2023). Decentralized Access Control for Secure Microservices Cooperation with Blockchain. ISA Trans..

[57] Cheng C., Yan B., Wang G. (2022). The Blockchain Based Access Control Scheme for the Internet of Things. Procedia Comput. Sci..

[58] Wang R., Wang X., Yang W., Yuan S., Guan Z. (2022). Achieving Fine-Grained and Flexible Access Control on Blockchain-Based Data Sharing for the Internet of Things. China Commun..

[59] Sun S., Chen S., Du R. (2020). Trusted and Efficient Cross-Domain Access Control System Based on Blockchain. Scientific Programming.

[60] Yang C., Tan L., Shi N., Xu B., Cao Y., Yu K. (2020). AuthPrivacyChain: A Blockchain-Based Access Control Framework with Privacy Protection in Cloud. IEEE Access.

[61] Schubert M.A., Pagel S., von Korflesch H.F.O. Success Factors in Secure Software Development of Cloud Applications in Germany: A Qualitative-Explorative Expert Study. Proceedings of the 56th Hawaii International Conference on System Sciences.

[62] Vakhula O., Kurii Y., Opirskyy I., Susukailo V. Security-as-Code Concept for Fulfilling ISO/IEC 27001:2022 Requirements. Proceedings of the Workshop on Cybersecurity Providing in Information and Telecommunication Systems (CPITS 2024).

[63] Kumar R., Goyal R. (2020). Modeling Continuous Security: A Conceptual Model for Automated DevSecOps Using Open-Source Software over Cloud (ADOC). Comput. Security.

[64] Kumar R., Goyal R. (2021). When Security Meets Velocity: Modeling Continuous Security for Cloud Applications Using Devsecops. Lect. Notes Data Eng. Commun. Technol..

[65] Dasher G., Envid I., Calder B. (2022). Architectures for Protecting Cloud Data Planes. arXiv.

[66] Jararweh Y. (2020). Enabling Efficient and Secure Energy Cloud Using Edge Computing and 5G. J. Parallel Distrib. Computing.

[67] Ali B., Gregory M.A., Li S. (2021). Multi-Access Edge Computing Architecture, Data Security and Privacy: A Review. IEEE Access.

[68] Telang T. (2023). Cloud-Native Application Development. Beginning Cloud Native Development with MicroProfile, Jakarta EE, and Kubernetes.

[69] Peng K., Zhao B., Bilal M., Xu X., Nayyar A. (2023). QoS-Aware Cloud-Edge Collaborative Micro-Service Scheduling in the IIoT. HCIS.

[70] Bhardwaj A., Benson T.A. (2022). Kubeklone: A Digital Twin for Simulating Edge and Cloud Microservices. HCIS.

[71] Malawski M., Gajek A., Zima A., Balis B., Figiela K. (2020). Serverless Execution of Scientific Workflows: Experiments with Hyperflow, Aws Lambda and Google Cloud Functions. Future Gener. Comput. Syst..

[72] Venugopal M.V.L.N., Reddy C.R.K. (2021). Serverless through Cloud Native Architecture. Int. J. Eng. Res. Technol..

[73] Mao Y., Fu Y., Gu S., Vhaduri S., Cheng L., Liu Q. (2020). Resource Management Schemes for Cloud-Native Platforms with Computing Containers of Docker and Kubernetes. Int. J. Eng. Res. Technol..

[74] Abernathey R.P., Augspurger T., Banihirwe A., Blackmon-Luca C.C., Crone T.J., Gentemann C.L., Hamman J.J., Henderson N., Lepore C., McCaie T.A. (2021). Cloud-Native Repositories for Big Scientific Data. Comput. Sci. Eng..

[75] Olave D. (2023). Towards Cloud-Native Context-Aware Autonomous Robotics for IoT Applications. Master’s Thesis.

[76] Kim T.W., Azzaoui A.E., Koh B., Kim J., Park J.H. (2022). A Secret Sharing-Based Distributed Cloud System for Privacy Protection. Hum. Centric Comput. Inf. Sci..

[77] Lim J. (2023). Versatile Cloud Resource Scheduling Based on Artificial Intelligence in Cloud-Enabled Fog Computing Environments. Hum. Centric Comput. Inf. Sci..

[78] Zheng P., Jiang Z., Wu J., Zheng Z. (2023). Blockchain-Based Decentralized Application: A Survey. IEEE Open J. Comput. Soc..

[79] Kumar R., Gupta S., Wang H., Kumari C.S., Korlam S.S.V.P. (2023). From Efficiency to Sustainability: Exploring the Potential of 6G for a Greener Future. Sustainability.

[80] Vinod S., Vimal V.R., Selvanayaki S. Screen Recording and Sharing over the Cloud Platform For Remote Teams And Cross-Functional Teams. Proceedings of the 2023 International Conference on Research Methodologies in Knowledge Management, Artificial Intelligence and Telecommunication Engineering (RMKMATE).

[81] Shridhar A., Nadig D. Heuristic-Based Resource Allocation for Cloud-Native Machine Learning Workloads. Proceedings of the 2022 IEEE International Conference on Advanced Networks and Telecommunications Systems (ANTS).

[82] Kosińska J., Baliś B., Konieczny M., Malawski M., Zieliński S. (2023). Toward the Observability of Cloud-Native Applications: The Overview of the State-of-the-Art. IEEE Access.

[83] Gao K., Sun C., Wang S., Li D., Zhou Y., Liu H.H., Zhu L., Zhang M., Deng X., Zhou C. (2023). Buffer-Based High-Coverage and Low-Overhead Request Event Monitoring in the Cloud. IEEE/ACM Trans. Netw..

[84] Kratzke N. (2022). Cloud-Native Observability: The Many-Faceted Benefits of Structured and Unified Logging—A Multi-Case Study. Future Internet.

[85] Sultan M., Rajaratnam D., Patel K. Enterprise Architecture Approach to Build API Economy. Proceedings of the 2022 International Conference on Computer Science and Software Engineering (CSASE).

[86] Al-Marsy A., Chaudhary P., Rodger J.A. (2021). A Model for Examining Challenges and Opportunities in Use of Cloud Computing for Health Information Systems. Appl. Syst. Innov..

[87] Cultrera F. (2022). A Performance Analysis of Mesh Models for Cloud-Based Workflows. Master’s Thesis.

[88] Chernyshev M., Baig Z., Zeadally S. (2021). Cloud-Native Application Security: Risks, Opportunities, and Challenges in Securing the Evolving Attack Surface. Computer.

[89] Christian J., Paulino L., de Sá A.O. (2022). A Low-Cost and Cloud Native Solution for Security Orchestration, Automation, and Response. Lecture Notes in Computer Science (Including Subseries Lecture Notes in Artificial Intelligence and Lecture Notes in Bioinformatics).

[90] Sisto S.P.R., Borgogni F., Casciaro L. (2023). Policy as Code, How to Automate Cloud Compliance Verification with Open-Source Tools. Ph.D. Thesis.

[91] Pandya S., Guha Thakurta R. (2022). Hands-on Infrastructure as Code with Hashicorp Terraform. Introduction to Infrastructure as Code.

[92] Marie-Magdelaine N. (2021). Observability and Resources Managements in Cloud-Native Environnements. Ph.D. Thesis.

[93] Jeon J., Jeong B., Jeong Y. (2024). PreVA: Predictive Vertical Autoscaler Using Multi Bi-GRU for Sustainable Cloud-Native Computing. Hum. Centric Comput. Inf. Sci..

[94] Chalishhafshejani S., Pham B., Jaatun M. Automating Security in a Continuous Integration Pipeline. Proceedings of the 7th International Conference on Internet of Things, Big Data and Security.

[95] Sankaran A., Datta P., Bates A. Workflow Integration Alleviates Identity and Access Management in Serverless Computing. Proceedings of the ACSAC’20: Proceedings of the 36th Annual Computer Security Applications Conference.

[96] Talluri S. (2024). Saviynt Meets GCP: A Deep Dive into Integrated IAM for Modern Cloud Security. J. Inf. Secur..

[97] Berkovich S., Kam J., Wurster G. {UBCIS}: Ultimate Benchmark for Container Image Scanning. Proceedings of the 13th USENIX Workshop on Cyber Security Experimentation and Test (CSET 20).

[98] Subramaniam K. (2022). Cloud Network Security: A Survey on Threats, Existing Solutions and the Path Forward.

[99] Kumar M., Jeni V. Web Application Security on Top of Public Cloud. Proceedings of the 022 Second International Conference on Interdisciplinary Cyber Physical Systems (ICPS).

[100] Lin Y., Xie Z., Chen T., Cheng X., Wen H. (2024). Image Privacy Protection Scheme Based on High-Quality Reconstruction DCT Compression and Nonlinear Dynamics. Expert Syst. Appl..

[101] Yang B., Zhang F., Khan S.U. An Encryption-as-a-Service Architecture on Cloud Native Platform. Proceedings of the 2021 International Conference on Computer Communications and Networks (ICCCN).

[102] German K., Ponomareva O. An Overview of Container Security in a Kubernetes Cluster. Proceedings of the 2023 IEEE Ural-Siberian Conference on Biomedical Engineering, Radioelectronics and Information Technology (USBEREIT).

[103] Kamruzzaman A., Ismat S., Brickley J.C., Liu A., Thakur K. A Comprehensive Review of Endpoint Security: Threats and Defenses. Proceedings of the 2022 International Conference on Cyber Warfare and Security (ICCWS).

[104] Kanerva S. (2024). Lateral Movement Restriction in Advanced DevOps Environments. Master’s Thesis.

[105] Henriques J., Caldeira F., Cruz T., Simões P. (2024). A Survey on Forensics and Compliance Auditing for Critical Infrastructure Protection. IEEE Access.

[106] Arif T., Jo B., Kang J., Park J.H. (2025). Blockchain-Enabled IDPS and Federated Learning for Enhancing CPS Security Against Advanced Persistent Threats in Zero Trust Architectures. Hum. Centric Comput. Inf. Sci..

[107] Ammi M., Adedugbe O., Alharby F.M., Benkhelifa E. (2022). Leveraging a Cloud-Native Architecture to Enable Semantic Interconnectedness of Data for Cyber Threat Intelligence. Clust. Comput..

[108] Nour B., Pourzandi M., Debbabi M. (2023). A Survey on Threat Hunting in Enterprise Networks. IEEE Commun. Surv. Tutorials.

